# A gastric cancer LncRNAs model for MSI and survival prediction based on support vector machine

**DOI:** 10.1186/s12864-019-6135-x

**Published:** 2019-11-13

**Authors:** Tao Chen, Cangui Zhang, Yingqiao Liu, Yuyun Zhao, Dingyi Lin, Yanfeng Hu, Jiang Yu, Guoxin Li

**Affiliations:** 1 Department of General Surgery, Nanfang Hospital, Southern Medical University, Guangdong Provincial Engineering Technology Research Center of Minimally Invasive Surgery, No.1838, North Guangzhou Avenue, Guangzhou, 510515 Guangdong Province China; 2 School of Biomedical Engineering, Southern Medical University, Guangzhou, 510515 Guangdong Province China

**Keywords:** Gastric cancer, Stomach adenocarcinoma, LncRNA, MSI, SVM, Prognosis

## Abstract

**Background:**

Recent studies have shown that long non-coding RNAs (lncRNAs) play a crucial role in the induction of cancer through epigenetic regulation, transcriptional regulation, post-transcriptional regulation and other aspects, thus participating in various biological processes such as cell proliferation, differentiation and apoptosis. As a new nova of anti-tumor therapy, immunotherapy has been shown to be effective in many tumors of which PD-1/PD-L1 monoclonal antibodies has been proofed to increase overall survival rate in advanced gastric cancer (GC). Microsatellite instability (MSI) was known as a biomarker of response to PD-1/PD-L1 monoclonal antibodies therapy. The aim of this study was to identify lncRNAs signatures able to classify MSI status and create a predictive model associated with MSI for GC patients.

**Methods:**

Using the data of Stomach adenocarcinoma from The Cancer Genome Atlas (TCGA), we developed and validated a lncRNAs model for automatic MSI classification using a machine learning technology – support vector machine (SVM). The C-index was adopted to evaluate its accuracy. The prognostic values of overall survival (OS) and disease-free survival (DFS) were also assessed in this model.

**Results:**

Using the SVM, a lncRNAs model was established consisting of 16 lncRNA features. In the training cohort with 94 GC patients, accuracy was confirmed with AUC 0.976 (95% CI, 0.952 to 0.999). Veracity was also confirmed in the validation cohort (40 GC patients) with AUC 0.950 (0.889 to 0.999). High predicted score was correlated with better DFS in the patients with stage I-III and lower OS with stage I-IV.

**Conclusion:**

This study identify 16 LncRNAs signatures able to classify MSI status. The correlation between lncRNAs and MSI status indicates the potential roles of lncRNAs interacting in immunotherapy for GC patients. The pathway of these lncRNAs which might be a target in PD-1/PD-L1 immunotherapy are needed to be further study.

## Background

The human genome contains thousands of long non-coding RNAs (lncRNAs), but only a few of them had been discovered their specific biological functions and biochemical mechanisms [1]. Recently a famous RNA NKILA, a NF-κB-interacting lncRNA, was demonstrated to promote tumor immune evasion by sensitizing T cells to activation-induced cell death [1, 2]. This indicated lncRNAs had values to be further studied in malignant tumor. Application of the lncRNAs as therapeutic targets and diagnostic markers is a potential progress [3].

Meanwhile, microsatellite instability (MSI) is characterized by high degree of polymorphism in microsatellite lengths due to deficiency in mismatch repair (MMR) system. It is a potential biomarker which can be reflected in gastric cancer (GC) patients with microsatellite instability-high (MSI-H) achieve superior responses to PD-1 antibody [4]. Significant difference in prognosis can be seen with different MSI state [5]. LncRNAs data analysis done by TANRIC showed there might be a correlation existed in lncRNAs and MSI [6]. However, numerous lncRNAs contributing to MSI still remain unclear and the mechanisms associated with MSI are needed to be discovered. Hence, we established and validated an lncRNAs model based on a machine learning technology – support vector machine (SVM) [7] for MSI prediction using the data of The Cancer Genome Atlas (TCGA). The prognostic value of this model was also evaluated in this study.

## Methods

### Search and collection of gastric cancer (GC) lncRNAs expression series

To ensure RNA transcript profiling data only contained lncRNAs, the data were download from TANRIC [6], which is an open-access resource for interactive exploration of lncRNAs in cancer. It characterizes the expression profiles of lncRNAs in large patient cohorts of 20 cancer types including TCGA, CCLE and other independent data cohorts. The data of Stomach adenocarcinoma (STAD) were collected for analysis in our study.

### Collection of clinical data

**Fig. 1:**
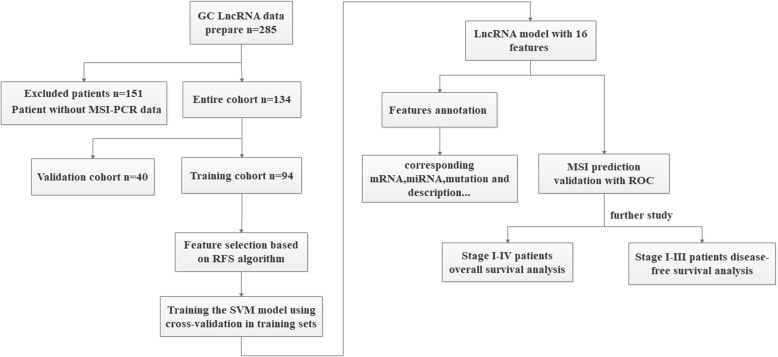
Flow chart of data collection and analysis

The clinical data of these series were obtained from TCGA [8]. Microsatellite instability-Polymerase Chain Reaction (MSI-PCR) data were obtained from R package “TCGAbiolinks”. Sample without MSI-PCR statistic was excluded from both training and validation cohorts (Fig. 1).

### Random grouping method

The feature data of all samples were normalized by the linear function normalization method.

The range of each dimension feature was limited to (0,1). The patients were assigned randomly in accordance with the ratio 7:3 to training cohort (94 patients) and validation cohort (40 patients) (Fig. 1).

### Search the best combination of support vector machines (SVM) model parameters

The Principal Component Analysis (PCA) algorithm is used on the normalized training cohort data. The PCA algorithm was conducted with MATLAB (version 2018a). Features which can reflect 95% information of the whole cohort were selected [9]. Support Vector Machines (SVM), introduced by Vapnik [7], is used for data classification and function approximation. SVM was conducted with MATLAB (version 2018a) using “LIBSVM” package. Parameter c was defined as 2; g was defined as 0.0884. To find out the best SVM model parameters (C and γ) combination with the highest average accuracy, cross-validation and grid search method were apply on the training cohort.

### Feature selection and model development

Relief forward selection algorithm (RFS) was adopted for feature selection. RFS combines ReliefF with a forward selection algorithm to handle the problem of feature (QC) redundancy [10]. RFS wad conducted with MATLAB (version 2018a). The original feature set contains a great deal of redundant and irrelevant features, which leads to model over-fitting. Feature selection is required to suppress over-fitting. Relief is a filter operator for feature selection. Relief design related statistics to measure the importance of features. This statistic is a vector, each component corresponds to an initial feature, and the importance of the feature subset depends on the sum of the relevant statistics reflected by each feature in the subset. Relief algorithm was used in the training cohort to obtain the sorted feature cohort represented by feature relief (FR). The parameter k, which means the k nearest neighbor samples, was defined as 10. Executed after forward selection steps, according to the order of the FR, starting from the first characteristic, will make them separately to improve the performance of classifiers features added to the sub cohort, and will be the candidate feature subset as SVM model [11, 12]. The input to train classification model, and through the AUC value to evaluate the prediction performance is good or bad, has the highest AUC value candidate feature subset will serve as the optimal features for the model development (Fig. 1).

### Performance assessment of lncRNAs model

The accuracy of MSI prediction in the lncRNAs model was verified with C-index. We assessed the prognostic accuracy of this model in the whole cohort using time-dependent receiver operator characteristics (ROC) analysis at different follow-up times (2, 3, 5 years). The patients were classified in to high and low risk score groups. The thresholds of classification were identified by using X-title [13]. The patients with clinical stage I-III and I-IV were used for DFS and OS analysis, respectively. We evaluated the potential association of the lncRNAs model with DFS and OS by using Kaplan-Meier survival method.

### Statistical analysis

Statistical analysis was conducted with R software (version 3.5.1; http://www.Rproject.org). Covariates balanced between MSI positive and negative patients statistic analysis were conducted with IBM SPSS Statistic software (version 22.0). Logistic regression was complete with R studio. C-index was done with “survival” package. Time dependent ROC analysis was done with “timeROC” package. ROC was analyzed with “pROC” package. Survival analysis was completed with “survival” package and “survminer” package. A two-sided *P* value < 0·05 was considered significant.

### Signature analysis

The correlated somatic mutation with LncRNAs signatures and the correlating mRNA and miRNA was based on the analysis results from TANRIC [14].

## Result

### Training and validation cohort preparation

GC lncRNAs data were downloaded from the publicly available TANRIC database containing 285 tumor samples and 33 normal samples. The data included 12,727 lncRNAs in total. The corresponding clinical data were obtained from TCGA database and MSI-PCR was obtained from R package. Patients without MSI-PCR were excluded in this study. The 134 patients were randomly assigned in 7:3 to the training cohort and validation cohort. In the training cohort, 94 patients were included, two of which were without full clinical data. In the validation cohort, 40 patients were included. Patient characteristics in the study are given in Additional file 1: Table S1. The age and sex covariates are balanced between MSI positive and negative patients (*P* > 0.05). (Additional file 4: Table S4**).**

### Development and assessment of lncRNAs model

Ten folds cross-validation was used to search the best combination of SVM model parameters: C and γ in the training cohort. The range of C was limited in (2^− 4^, 2^8^) and γ was limited in (2^− 8^, 2^6^).

**Fig. 2:**
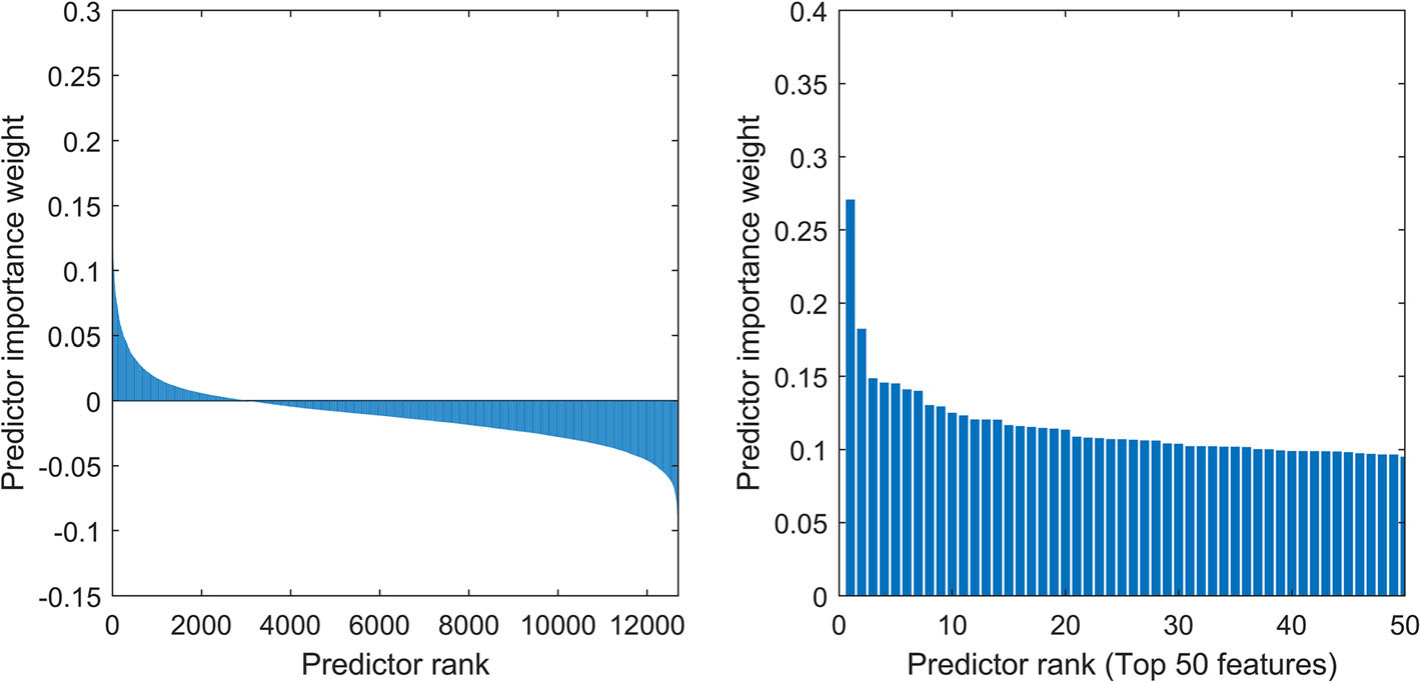
Feature selection using Relief forward selection algorithm (RFS). The left graph is the weight ranking of all the features. The right graph is the weight ranking of the first 50 features of the weight size

In the training cohort, Relief algorithm was used to obtain the sorted lncRNAs represented by FR [15]. The weight ordering of lncRNAs was shown in Fig. 2. As can be seen from the figure, when the loop reached the position of the blue dotted line, the AUC value of the feature subset had reached a high level and the AUC value didn’t not change much when the new feature was added. Therefore, considering the complexity of the model, the corresponding feature subset (including 16 features) at the position of the dotted line were selected as the optimal features. The lncRNAs model was developed included the 16 optimal features (Table 1).

**Table 1:** LncRNAs significantly associated with the MSI in the training cohort

Gene stable ID version	Gene stable ID	Transcript stable ID	Gene name	Gene description	Transcript name	*P*-value
ENSG00000229175.1	ENSG00000229175	ENST00000427918	LINC00382	long intergenic non-protein coding RNA 382 [Source:HGNC Symbol;Acc:HGNC:42709]	LINC00382-201	0.0901
ENSG00000231125.2	ENSG00000231125	ENST00000457162	AF129075.1	novel transcript, sense intronic to CCT8	AF129075.1-201	0.091
ENSG00000231394.1	ENSG00000231394	ENST00000449899	AC099681.2	novel transcript	AC099681.2-201	0.009
ENSG00000236457.1	ENSG00000236457	ENST00000413674	AC090617.1	novel transcript, sense intronic to SMG6	AC090617.1-201	0.005
ENSG00000237200.1	ENSG00000237200	ENST00000438551	ZBTB40-IT1	ZBTB40 intronic transcript 1 [Source:HGNC Symbol;Acc:HGNC:41493]	ZBTB40-IT1-201	< 0.001
ENSG00000237923.1	ENSG00000237923	ENST00000442852	LINC02570	long intergenic non-protein coding RNA 2570 [Source:HGNC Symbol;Acc:HGNC:39766]	LINC02570-206	0.132
ENSG00000253567.1	ENSG00000253567	ENST00000523935	AC025871.1	novel transcript	AC025871.1-201/AC025871.1-202	0.104
ENSG00000261117.1	ENSG00000261117	ENST00000569860	AC009486.1	novel transcript	AC009486.1-201	0.045
ENSG00000261501.1	ENSG00000261501	ENST00000567769	AC079341.1	novel transcript	AC079341.1-201	0.219
ENSG00000263904.1	ENSG00000263904	ENST00000581134	AC015563.1	novel transcript, sense intronic to FAM59A	AC015563.1-201	0.15
ENSG00000272562.1	ENSG00000272562	ENST00000609423	AL512343.2	novel transcript, antisense to H3F3A	AL512343.2-201	0.010
ENSG00000175061.13	ENSG00000175061	ENST00000484836	LRRC75A-AS1	LRRC75A antisense RNA 1	–	< 0.001
ENSG00000221571.2	ENSG00000221571	ENST00000609276	RNU6ATAC35P	RNA, U6atac small nuclear 35, pseudogene	–	0.005
ENSG00000226673.1	ENSG00000226673	ENST00000427276	LINC01108	long intergenic non-protein coding RNA 1108	–	0.253
ENSG00000232732.5	ENSG00000232732	ENST00000601136	AC097717.1	novel transcript	–	0.045
ENSG00000251538.1	ENSG00000251538	ENST00000511194	LINC02201	long intergenic non-protein coding RNA 2201	–	0.109

“-” Not reported. *P*-value: logistic regression of each lncRNA and MSI state

**Fig. 3:**
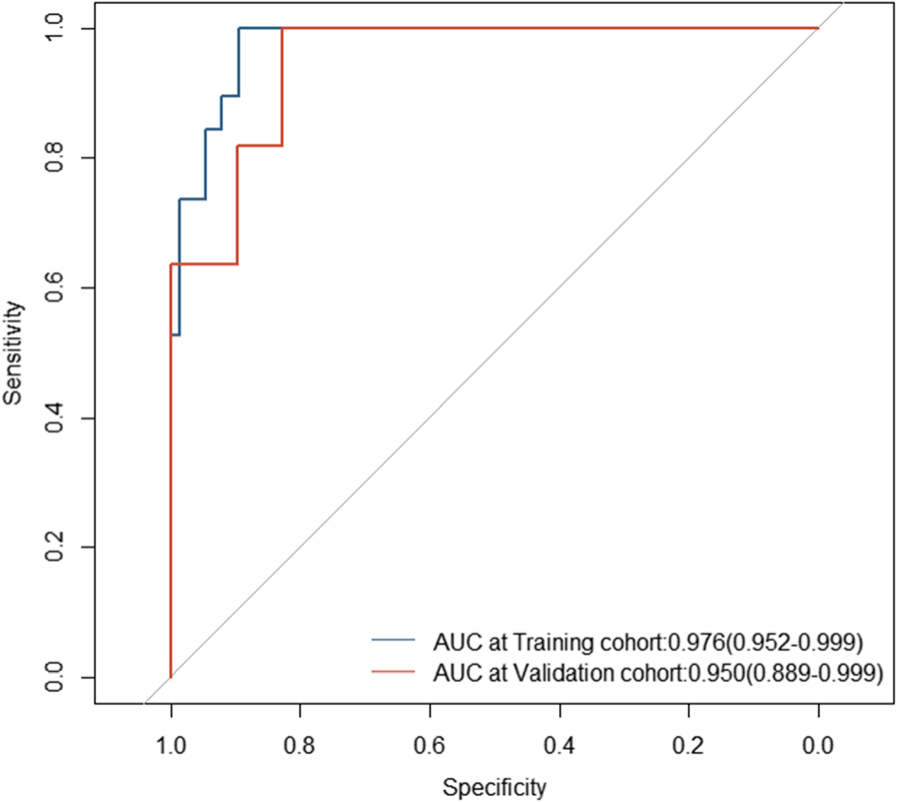
The lncRNAs model measured by receiver–operating characteristic (ROC) curves in the training cohort and validation cohort

**Fig. 4:**
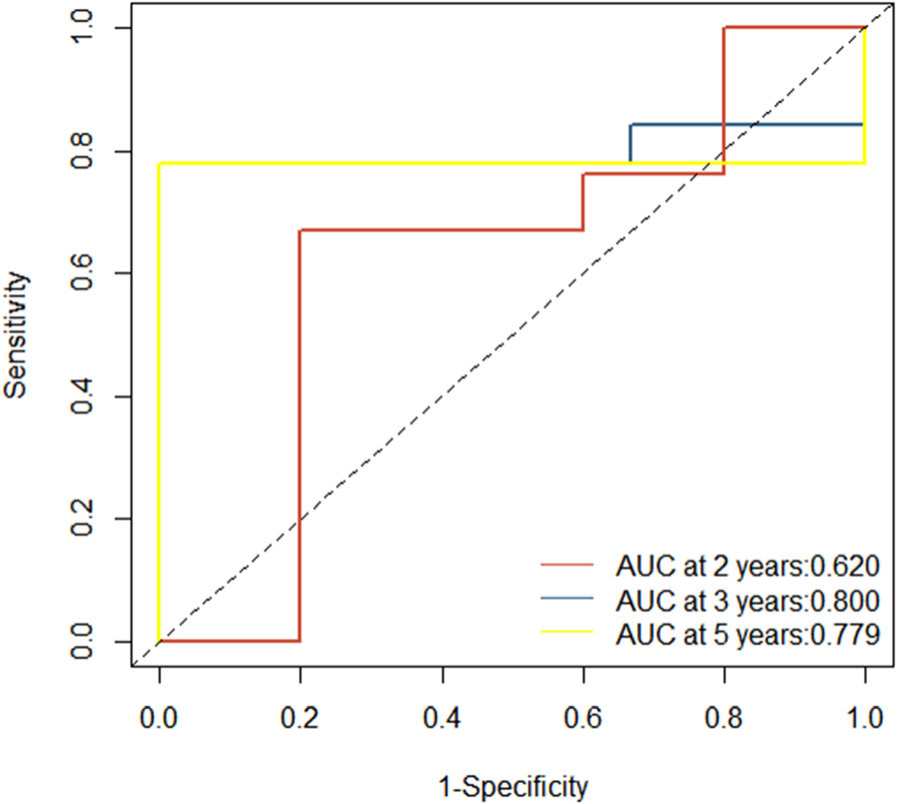
The lncRNAs model measured by time-dependent receiver–operating characteristic (ROC) curves at 2, 3, 5 years

**Fig. 5:**
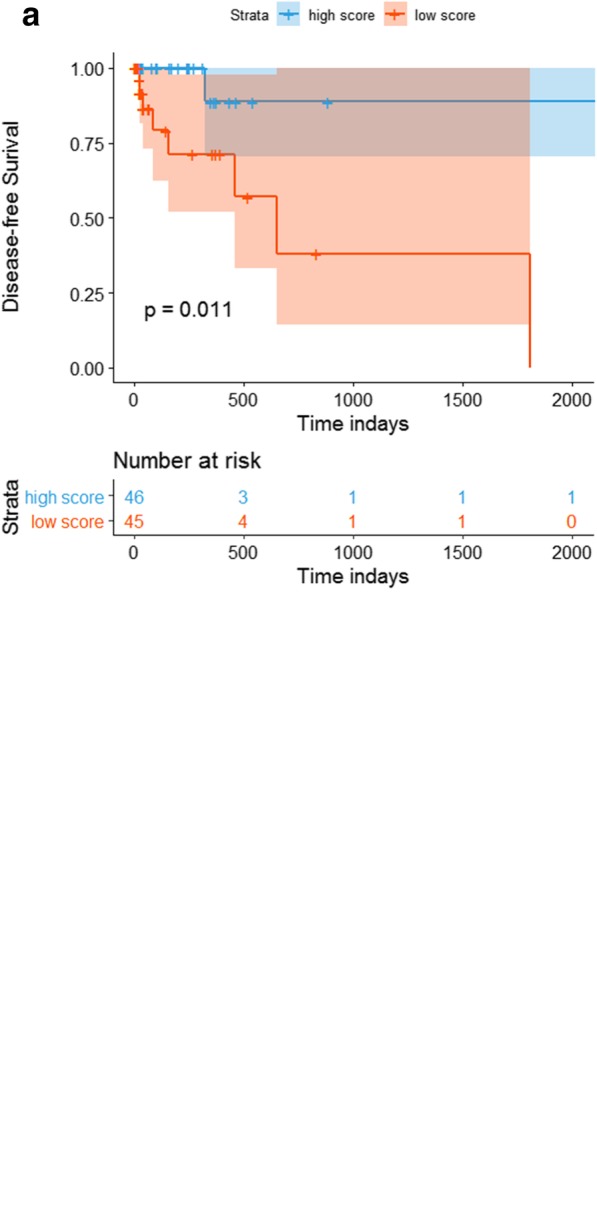
Survival impact of the lncRNAs model. **a** Kaplan–Meier curves for disease-free survival (DFS) by the lncRNAs model’s scores with patients with stage I-III. **b** Kaplan–Meier curves for overall survival (OS) by the lncRNAs model’s scores with patients with stage I-IV

The AUC for the lncRNAs model’s sensitivity was 0.976 (95% CI, 0.952 to 0.999) for the training cohort, which was confirmed to be 0.950 (0.889 to 0.999) in the validation cohort. Both training cohort and validation were via bootstrapping validation (Fig. 3). The AUC at 2, 3, 5 years were 0.620 (95% CI, 0.234 to 0.999), 0.800 (0.495 to 0.999), 0.779 (0.463 to 0.999), respectively (Fig. 4). The patients were assigned to a high- or low- score group using the cut-off value obtained from the entire cohort (DFS, 0.089; OS, 0.183). The patients of clinical stage I-III with high- score had a significant higher DFS rate than the patients with low-score (*P* = 0.011). However, a higher OS rate was seen in the patients with low-score in clinical stage I-IV (*P* = 0.028) (Fig. 5a and b).

## Discussion

The lncRNAs model, a novel tool with satisfactory performance, was aimed at selecting lncRNAs of GC to further study MSI (Additional file 3). It can also be used as a method to predict MSI state with lncRNAs for immunotherapy. For the construction of the lncRNA model, 16 of 12,727 lncRNAs were selected to incorporate. Among these 16 lncRNAs, 8 of them can be individual predictors of MSI (all *P* < 0.05).

Survival analysis indicated the lncRNAs model also has prognostic value. For the reason that the limited samples of our cohort, patients were insufficient to assign to two group to verify the lncRNAs model after excluding the patients without complete clinical information, we choose to verify the prognostic value in the entire cohort. To date, some studies have demonstrated the association between MSI state and OS in GC patients [16], however, studies about the correlation between MSI state and DFS are rare. Our results demonstrated the lncRNAs model can predict prognosis of DFS in the clinical stage of I-III. Patients with high-score of the lncRNAs model, also regard as MSI-H, have better DFS compared with the patients with low-score regarded as MSS. But for the clinical stage of I-IV, patients with low-score have a better OS compared with the patients with high-score.

TNM stage was known as the prognostic criteria in GC. Compared with TNM stage ROC (AUC at 2, 3, 5 were 0.679, 0.545, 0.722), the LncRNAs model has a better prognostic performance. (Additional file 5: Figure S1**)** The AUC of MSI prognostic ROC at 2, 3, 5 were 0.618, 0.811, 0.811, similar performance with the LncRNAs model. (Additional file 6: Figure S2) Survival analysis illustrated MSI has no statistical significance. (Additional file 7: Figure S3) TNM method have no statistical significance in survival analysis when predicting stage I-IV patients overall survival or disease-free survival. (Additional file 8: Figure S4 and Additional file 9: Figure S5).

LncRNAs play crucial role in the pathogenesis of cancer and their dysfunctions are related to cancer development and progression, as reviewed in multiple reports [17, 18]. Differential analysis revealed that lncRNAs have correlation with somatic mutation (Additional file 3). Additional file 2: Table S2 showed the correlated somatic mutation of each lncRNA. LINC00382 is one of the optimal lncRNAs subset in our model. LINC00382 and TP53 had statistical significance with *P*-value< 0.05. TP53 was known as an anti-oncogene, and its mutation was proofed to be the most relevant with cancer while lncRNAs have been proofed to act as regulatory molecules to regulate P53 genes and cell cycle [19]. Previous study suggested that TP53 can be used as a biomarker of microsatellite, which verify the significance of lncRNA model and our lncRNAs [20]. Furthermore, the correlation between the lncRNAs and somatic mutation indicated a potential pathway existing to affect MSI and even the development of cancer. Moreover, significant somatic mutation could be seen in ASH1L. ASH1L, reported as an important role in modulating immune response and inflammation, has a correlation with lncRNA ZBTB40-IT1 (*P*-value< 0.05) which was also included in our model [21]. Correlation can be seen in ATM and ZBTB40-IT1. ATM plays a crucial role in DNA double-strain repairing, acting on cell-cycle checkpoint arrest (e.g., Chk1 and Chk2), DNA repair (BRCA1 and RAD51), and apoptosis (p53; ref. [15]) [22]. The somatic mutation in ATM was proofed to occur in GC [23]. High expression of ATM and MSI-H exhibited better prognosis of DFS and OS [24]. Both of these somatic mutations and our lncRNAs have correlation with MSI. For MSI is the most valuable immunomarker in PD-1/PD-L1 immunotherapy, emphasis is raised in this potential pathway which could be new targets in immunotherapy.

Compared with other conventional machine learning algorithms, the SVM algorithm greatly simplifies the complexity of computation because it uses the inner product kernel function instead of the nonlinear mapping to the high-dimensional space and better suited to manage classification based on high-dimensional data with a limited number of training cohort to select the most efficient of all available features [25, 26]. Previous studies have shown that single biomarker has limited prognostic value for GC [27–29]. At the same time, compared with the deep learning [30], the advanced algorithm of artificial intelligence (AI), SVM has better generalization ability than neural network in the classification of small samples, and the phenomenon of over-fitting is not easy after combining penalty term. Considering the limited samples in the study, SVM was selected for the model development instead of deep learning.

Relief-based Forward Selection Algorithm (RFS) has good performance in feature reduction. In this paper, we used this method as the result of the posterior experience to modify the grid search method repeatedly, which makes the final model have better prediction ability. Though not all these features had the highest predictive value, the accuracy of the model was the best.

The limitations should be acknowledged for our study. First, this study was based on publicly available data sets, and it was not possible to obtain all information needed for each patient. Recent studies revealed that patients with older age, female, Laurén histological type, mid/lower gastric location and lack of lymph node metastases have higher possibility of MSI-H [16, 31, 32]. Insufficient data was unable to verify these indexes in the study. Second, as all patients in this study were selected retrospectively, the potential bias relating to unbalanced clinical pathological features with treatment heterogeneity cannot be ignored. Further prospective studies are required to validate the results. Finally, we have no experimental data and lack information on the mechanism behind the signature lncRNAs, and experimental studies on these lncRNAs are greatly needed. Even so, our finding might provide certain reference value for further researches in the functional roles of these lncRNAs.

## Conclusions

This study concentrates on the correlation between lncRNAs and MSI and presents 16 feature lncRNAs with predictive value of MSI. Moreover, this lncRNAs model with different MSI states may acted as potential biomarkers for GC prognostication. Further study may focus on validation of our finding and functional pathways of MSI and these lncRNAs.

## Supplementary information

**Additional file 1: Table S1.** Characteristics of patients in the training and validation cohorts. Values in parentheses are percentages. *TNM eighth edition.

**Additional file 2: Table S2.** Relative somatic mutation of 16 feature lncRNAs. The table shows somatic mutation of 16 feature lncRNAs with *P*-value < 0.05. All data was downloaded from TANRIC.

**Additional file 3:** **Table S3.** Correlating mRNA and miRNA of 16 feature lncRNAs. The table shows correlating mRNA and miRNA of 16 feature lncRNAs with P-value < 0.05. All data was downloaded from TANRIC.

**Additional file 4: Table S4.** Characteristics of patients in the MSI-H and MSS cohorts.

**Additional file 5: Figure S1.** The TNM stage measured by time-dependent receiver–operating characteristic curves at 2, 3, 5 years.

**Additional file 6: Figure S2.** The MSI measured by time-dependent receiver–operating characteristic curves at 2, 3, 5 years.

**Additional file 7: Figure S3.** Survival impact of the MSI state. Kaplan–Meier curves for overall survival (OS) by the MSI state with patients with stage I-IV.

**Additional file 8: Figure S4.** Survival impact of the TNM stage. Kaplan–Meier curves for overall survival (OS) by the TNM stage with patients with stage I-IV.

**Additional file 9: Figure S5.** Survival impact of the TNM stage. Kaplan–Meier curves for disease-free survival (DFS) by the TNM stage with patients with stage I-IV.

## Data Availability

The lncRNAs expression data in this study can be found online at The Atlas of ncRNA in Cancer (https://ibl.mdanderson.org/tanric/_design/basic/download.html).

## Abbreviations

AUC: Area under curve
CI: Confidence interval
DFS: Disease free survival
FR: Feature relief
GC: Gastric cancer
lncRNAs: Long non-coding RNAs
MSI: Microsatellite instability
MSI-H: Microsatellite instability high
MSS: Microsatellite stable
OS: Overall survival
PCA: Principal component analysis
RFS: Relief forward selection algorithm
ROC: Receiver operating characteristic
SVM: Support vector machine
TCGA: The cancer genome atlas
timeROC: Time-dependent receiver operating characteristic
