# Supplementary material for: A gastric cancer LncRNAs model for MSI and survival prediction based on support vector machine
Source: BMC Genomics. 2019 Nov 13;20:846. doi: 10.1186/s12864-019-6135-x (PMC6854775; doi:10.1186/s12864-019-6135-x)
Supplement: Supplementary file 1 — Additional file 1: Table S1. Characteristics of patients in the training and validation cohorts. Values in parentheses are percentages. *TNM eighth edition. [file 12864_2019_6135_MOESM1_ESM.docx]

| **Table S1**. **Characteristics of patients in the training and validation cohorts** | | | | |
| --- | --- | --- | --- | --- |
|  | Training Cohort | | Validation Cohort | |
| Characteristic | MSI-H | MSS | MSI-H | MSS |
| Age(years), No (%) |  |  |  |  |
| 18-64 | 6(31.6) | 34(45.3) | 1(9.0) | 12(41.4) |
| >64 | 13（68.4） | 39(52.0) | 10(91.0) | 17(57.6) |
| Unknown | 0 | 2(2.6) | 0 | 0 |
| Gender, No. (%) |  |  |  |  |
| Male | 10(53.0) | 49(65.3) | 0 | 22(75.9) |
| Female | 9(47.0) | 26(34.7) | 11(100) | 7(24.1) |
| Tumour stage*, No (%) |  |  |  |  |
| I | 3(15.8) | 10(13.3) | 1(9.2) | 4(13.8) |
| II | 7(36.8) | 26(34.6) | 3(27.3) | 9(31.0) |
| III | 3(15.8) | 31(41.3) | 3(27.3) | 9(31.0) |
| IV | 1(5.3) | 5(6.8) | 2(18.1) | 2(6.8) |
| Unknown | 5(26.3) | 3(4.0) | 2(18.1) | 5(17.4) |

Values in parentheses are percentages. *TNM eighth edition.
