# Supplementary material for: A gastric cancer LncRNAs model for MSI and survival prediction based on support vector machine
Source: BMC Genomics. 2019 Nov 13;20:846. doi: 10.1186/s12864-019-6135-x (PMC6854775; doi:10.1186/s12864-019-6135-x)
Supplement: Supplementary file 2 — Additional file 2: Table S2. Relative somatic mutation of 16 feature lncRNAs. The table shows somatic mutation of 16 feature lncRNAs with P-value < 0.05. All data was downloaded from TANRIC. [file 12864_2019_6135_MOESM2_ESM.docx]

| **Table S2 Relative somatic mutation of 16 feature lncRNAs** | | | | | |
| --- | --- | --- | --- | --- | --- |
| Cancer | Annotation | Gene Symbol | Mutant Mean | Wildtype Mean | P-value |
| TCGA-STAD | ENSG00000237200.1 | FCGBP | -2.570 | -3.645 | <0.001 |
| TCGA-STAD | ENSG00000237200.1 | TRIO | -2.325 | -3.650 | <0.001 |
| TCGA-STAD | ENSG00000237200.1 | KCNH8 | -2.524 | -3.643 | 0.001 |
| TCGA-STAD | ENSG00000237200.1 | ZC3H13 | -2.223 | -3.654 | 0.002 |
| TCGA-STAD | ENSG00000237200.1 | ZEB2 | -2.323 | -3.650 | 0.003 |
| TCGA-STAD | ENSG00000237200.1 | ASH1L | -2.402 | -3.647 | 0.005 |
| TCGA-STAD | ENSG00000237200.1 | CENPF | -2.507 | -3.643 | 0.009 |
| TCGA-STAD | ENSG00000236457.1 | ANK2 | -15.559 | -8.736 | <0.001 |
| TCGA-STAD | ENSG00000236457.1 | ARFGEF1 | -14.198 | -8.810 | 0.009 |
| TCGA-STAD | ENSG00000236457.1 | ATP10A | -13.817 | -8.806 | 0.006 |
| TCGA-STAD | ENSG00000236457.1 | CIT | -14.178 | -8.811 | 0.009 |
| TCGA-STAD | ENSG00000236457.1 | CSMD1 | -13.056 | -8.592 | <0.001 |
| TCGA-STAD | ENSG00000236457.1 | CSMD2 | -13.213 | -8.765 | 0.005 |
| TCGA-STAD | ENSG00000236457.1 | CTNNA2 | -14.444 | -8.802 | 0.004 |
| TCGA-STAD | ENSG00000236457.1 | DDI1 | -14.514 | -8.799 | 0.003 |
| TCGA-STAD | ENSG00000236457.1 | GPR98 | -12.482 | -8.679 | 0.003 |
| TCGA-STAD | ENSG00000236457.1 | HERC1 | -14.054 | -8.777 | 0.002 |
| TCGA-STAD | ENSG00000236457.1 | KALRN | -13.953 | -8.782 | 0.003 |
| TCGA-STAD | ENSG00000236457.1 | KIAA0947 | -14.284 | -8.787 | 0.006 |
| TCGA-STAD | ENSG00000236457.1 | MACF1 | -12.843 | -8.756 | 0.006 |
| TCGA-STAD | ENSG00000236457.1 | MLL3 | -12.539 | -8.747 | 0.009 |
| TCGA-STAD | ENSG00000236457.1 | MUC16 | -11.148 | -8.628 | 0.010 |
| TCGA-STAD | ENSG00000236457.1 | OBSCN | -12.387 | -8.744 | 0.009 |
| TCGA-STAD | ENSG00000236457.1 | PCDHA11 | -14.678 | -8.793 | 0.001 |
| TCGA-STAD | ENSG00000236457.1 | PKD1L2 | -14.248 | -8.809 | 0.007 |
| TCGA-STAD | ENSG00000236457.1 | PXDN | -13.225 | -8.765 | 0.005 |
| TCGA-STAD | ENSG00000236457.1 | RERE | -14.197 | -8.751 | 0.001 |
| TCGA-STAD | ENSG00000236457.1 | REV3L | -13.757 | -8.790 | 0.007 |
| TCGA-STAD | ENSG00000236457.1 | RYR1 | -12.501 | -8.749 | 0.010 |
| TCGA-STAD | ENSG00000236457.1 | TRRAP | -12.363 | -8.732 | 0.009 |
| TCGA-STAD | ENSG00000236457.1 | TTN | -10.917 | -8.520 | 0.005 |
| TCGA-STAD | ENSG00000236457.1 | USH2A | -13.489 | -8.715 | 0.001 |
| TCGA-STAD | ENSG00000236457.1 | VPS13B | -12.771 | -8.775 | 0.008 |
| TCGA-STAD | ENSG00000261501.1 | ATM | -15.779 | -12.846 | 0.006 |
| TCGA-STAD | ENSG00000261501.1 | DOCK10 | -15.895 | -12.820 | 0.001 |
| TCGA-STAD | ENSG00000261501.1 | EPHA6 | -15.803 | -12.845 | 0.005 |
| TCGA-STAD | ENSG00000261501.1 | FMN2 | -15.917 | -12.830 | 0.001 |
| TCGA-STAD | ENSG00000261501.1 | FSHR | -15.854 | -12.832 | 0.002 |
| TCGA-STAD | ENSG00000261501.1 | REV3L | -15.895 | -12.820 | 0.001 |
| TCGA-STAD | ENSG00000261501.1 | RTTN | -15.722 | -12.838 | 0.009 |
| TCGA-STAD | ENSG00000261501.1 | UTRN | -15.989 | -12.804 | <0.001 |
| TCGA-STAD | ENSG00000272562.1 | PREX2 | -8.755 | -4.229 | 0.010 |
| TCGA-STAD | ENSG00000272562.1 | ZNF536 | -3.531 | -4.569 | 0.002 |
| TCGA-STAD | ENSG00000231394.1 | NBEA | -15.786 | -13.029 | 0.006 |
| TCGA-STAD | ENSG00000251538.1 | ACVR2A | -7.593 | -11.199 | 0.001 |
| TCGA-STAD | ENSG00000251538.1 | DOCK3 | -8.084 | -11.210 | 0.004 |
| TCGA-STAD | ENSG00000251538.1 | FHOD3 | -7.115 | -11.237 | <0.001 |
| TCGA-STAD | ENSG00000251538.1 | FLNA | -7.584 | -11.160 | 0.008 |
| TCGA-STAD | ENSG00000251538.1 | RYR1 | -8.542 | -11.213 | 0.008 |
| TCGA-STAD | ENSG00000251538.1 | TRIP12 | -8.006 | -11.157 | 0.008 |
| TCGA-STAD | ENSG00000231125.2 | TMEM132D | -4.035 | -3.019 | 0.003 |
| TCGA-STAD | ENSG00000237923.1 | MYH9 | -6.252 | -10.756 | 0.002 |
| TCGA-STAD | ENSG00000226673.1 | CBS | -7.188 | -8.513 | <0.001 |
| TCGA-STAD | ENSG00000226673.1 | FSHR | -12.685 | -8.297 | 0.010 |
| TCGA-STAD | ENSG00000226673.1 | PTPRM | -6.907 | -8.535 | 0.004 |
| TCGA-STAD | ENSG00000261117.1 | ACACA | -4.268 | -5.516 | 0.010 |
| TCGA-STAD | ENSG00000261117.1 | AKAP13 | -4.314 | -5.519 | 0.007 |
| TCGA-STAD | ENSG00000261117.1 | AKAP9 | -4.224 | -5.527 | 0.001 |
| TCGA-STAD | ENSG00000261117.1 | APOB | -4.202 | -5.558 | <0.001 |
| TCGA-STAD | ENSG00000261117.1 | ARFGEF1 | -4.395 | -5.512 | 0.007 |
| TCGA-STAD | ENSG00000261117.1 | ATM | -3.610 | -5.540 | <0.001 |
| TCGA-STAD | ENSG00000261117.1 | ATP10A | -4.214 | -5.523 | 0.002 |
| TCGA-STAD | ENSG00000261117.1 | ATRX | -4.244 | -5.517 | 0.002 |
| TCGA-STAD | ENSG00000261117.1 | BCOR | -4.447 | -5.510 | 0.005 |
| TCGA-STAD | ENSG00000261117.1 | CACNA1B | -4.272 | -5.516 | 0.008 |
| TCGA-STAD | ENSG00000261117.1 | CELSR1 | -4.061 | -5.540 | <0.001 |
| TCGA-STAD | ENSG00000261117.1 | CHD9 | -4.050 | -5.524 | 0.006 |
| TCGA-STAD | ENSG00000261117.1 | CMYA5 | -4.173 | -5.520 | 0.001 |
| TCGA-STAD | ENSG00000261117.1 | COL1A2 | -4.283 | -5.516 | 0.006 |
| TCGA-STAD | ENSG00000261117.1 | CTNND2 | -4.377 | -5.542 | 0.006 |
| TCGA-STAD | ENSG00000261117.1 | CUBN | -4.345 | -5.549 | 0.009 |
| TCGA-STAD | ENSG00000261117.1 | DNAH11 | -4.082 | -5.555 | <0.001 |
| TCGA-STAD | ENSG00000261117.1 | DNAH2 | -4.235 | -5.522 | 0.004 |
| TCGA-STAD | ENSG00000261117.1 | DNAH7 | -4.425 | -5.547 | 0.008 |
| TCGA-STAD | ENSG00000261117.1 | DOCK9 | -4.359 | -5.521 | 0.003 |
| TCGA-STAD | ENSG00000261117.1 | EDNRB | -4.409 | -5.511 | 0.006 |
| TCGA-STAD | ENSG00000261117.1 | EPB41L3 | -4.155 | -5.530 | 0.002 |
| TCGA-STAD | ENSG00000261117.1 | EPHA6 | -3.500 | -5.544 | 0.001 |
| TCGA-STAD | ENSG00000261117.1 | FSHR | -4.041 | -5.530 | 0.007 |
| TCGA-STAD | ENSG00000261117.1 | GFOD1 | -3.691 | -5.537 | 0.001 |
| TCGA-STAD | ENSG00000261117.1 | GPR98 | -4.535 | -5.559 | 0.002 |
| TCGA-STAD | ENSG00000261117.1 | GRIA2 | -4.083 | -5.528 | 0.001 |
| TCGA-STAD | ENSG00000261117.1 | GRM5 | -4.305 | -5.515 | 0.004 |
| TCGA-STAD | ENSG00000261117.1 | GTF3C1 | -4.047 | -5.530 | 0.002 |
| TCGA-STAD | ENSG00000261117.1 | KIAA1109 | -4.028 | -5.558 | <0.001 |
| TCGA-STAD | ENSG00000261117.1 | LAMA2 | -4.333 | -5.523 | 0.004 |
| TCGA-STAD | ENSG00000261117.1 | LRRK2 | -4.246 | -5.531 | 0.004 |
| TCGA-STAD | ENSG00000261117.1 | MDC1 | -4.064 | -5.524 | 0.002 |
| TCGA-STAD | ENSG00000261117.1 | MEGF6 | -3.900 | -5.530 | <0.001 |
| TCGA-STAD | ENSG00000261117.1 | MUC17 | -4.397 | -5.537 | 0.004 |
| TCGA-STAD | ENSG00000261117.1 | NEB | -4.060 | -5.546 | <0.001 |
| TCGA-STAD | ENSG00000261117.1 | NID1 | -4.207 | -5.523 | 0.005 |
| TCGA-STAD | ENSG00000261117.1 | NRP2 | -3.907 | -5.529 | 0.003 |
| TCGA-STAD | ENSG00000261117.1 | NRXN1 | -3.953 | -5.528 | <0.001 |
| TCGA-STAD | ENSG00000261117.1 | NUP98 | -4.020 | -5.525 | 0.008 |
| TCGA-STAD | ENSG00000261117.1 | OBSCN | -4.503 | -5.546 | 0.003 |
| TCGA-STAD | ENSG00000261117.1 | PCDHB12 | -4.039 | -5.525 | <0.001 |
| TCGA-STAD | ENSG00000261117.1 | PDZD2 | -4.294 | -5.543 | 0.004 |
| TCGA-STAD | ENSG00000261117.1 | PIK3CA | -4.276 | -5.573 | <0.001 |
| TCGA-STAD | ENSG00000261117.1 | POLQ | -3.838 | -5.532 | <0.001 |
| TCGA-STAD | ENSG00000261117.1 | PTPRM | -4.470 | -5.517 | 0.002 |
| TCGA-STAD | ENSG00000261117.1 | RTTN | -3.938 | -5.534 | 0.003 |
| TCGA-STAD | ENSG00000261117.1 | RYR2 | -4.359 | -5.580 | 0.001 |
| TCGA-STAD | ENSG00000261117.1 | SACS | -4.286 | -5.543 | 0.002 |
| TCGA-STAD | ENSG00000261117.1 | SCN5A | -4.186 | -5.519 | 0.007 |
| TCGA-STAD | ENSG00000261117.1 | SIPA1L1 | -4.050 | -5.535 | 0.001 |
| TCGA-STAD | ENSG00000261117.1 | SPEG | -4.035 | -5.536 | 0.003 |
| TCGA-STAD | ENSG00000261117.1 | SPTA1 | -4.505 | -5.546 | 0.007 |
| TCGA-STAD | ENSG00000261117.1 | SPTBN4 | -4.445 | -5.530 | 0.009 |
| TCGA-STAD | ENSG00000261117.1 | TDRD6 | -3.899 | -5.542 | <0.001 |
| TCGA-STAD | ENSG00000261117.1 | TIAM1 | -3.906 | -5.535 | <0.001 |
| TCGA-STAD | ENSG00000261117.1 | UNC13B | -4.212 | -5.523 | 0.009 |
| TCGA-STAD | ENSG00000261117.1 | UNC13C | -3.913 | -5.547 | <0.001 |
| TCGA-STAD | ENSG00000232732.5 | PIK3CA | -9.906 | -7.284 | 0.009 |
| TCGA-STAD | ENSG00000221571.2 | ABCC9 | -4.896 | -7.902 | <0.001 |
| TCGA-STAD | ENSG00000221571.2 | AHNAK2 | -5.314 | -7.922 | 0.006 |
| TCGA-STAD | ENSG00000221571.2 | ARFGEF1 | -3.956 | -7.914 | <0.001 |
| TCGA-STAD | ENSG00000221571.2 | ARID1A | -4.983 | -7.998 | <0.001 |
| TCGA-STAD | ENSG00000221571.2 | ATRX | -4.244 | -7.904 | <0.001 |
| TCGA-STAD | ENSG00000221571.2 | BOD1L1 | -4.443 | -7.897 | <0.001 |
| TCGA-STAD | ENSG00000221571.2 | BSN | -4.156 | -7.935 | <0.001 |
| TCGA-STAD | ENSG00000221571.2 | CACNA1B | -3.923 | -7.916 | <0.001 |
| TCGA-STAD | ENSG00000221571.2 | CACNA1E | -4.530 | -7.956 | <0.001 |
| TCGA-STAD | ENSG00000221571.2 | CBS | -4.445 | -7.897 | <0.001 |
| TCGA-STAD | ENSG00000263904.1 | CAMTA1 | -13.813 | -8.974 | 0.008 |
| TCGA-STAD | ENSG00000263904.1 | CPAMD8 | -13.109 | -8.923 | 0.008 |
| TCGA-STAD | ENSG00000263904.1 | CTNNA2 | -14.701 | -8.942 | 0.001 |
| TCGA-STAD | ENSG00000263904.1 | GPR98 | -12.435 | -8.841 | 0.005 |
| TCGA-STAD | ENSG00000263904.1 | LAMA1 | -13.436 | -8.939 | 0.007 |
| TCGA-STAD | ENSG00000263904.1 | NRP2 | -13.865 | -8.972 | 0.007 |
| TCGA-STAD | ENSG00000263904.1 | RERE | -13.387 | -8.941 | 0.008 |
| TCGA-STAD | ENSG00000263904.1 | SIPA1L1 | -13.874 | -8.936 | 0.006 |
| TCGA-STAD | ENSG00000263904.1 | XIST | -12.781 | -8.913 | 0.010 |
| TCGA-STAD | ENSG00000229175.1 | DNAH11 | -16.283 | -14.950 | 0.002 |
| TCGA-STAD | ENSG00000229175.1 | DNAH5 | -16.224 | -14.939 | 0.007 |
| TCGA-STAD | ENSG00000229175.1 | DYNC2H1 | -16.262 | -14.956 | 0.004 |
| TCGA-STAD | ENSG00000229175.1 | KIAA1109 | -16.283 | -14.950 | 0.002 |
| TCGA-STAD | ENSG00000229175.1 | NALCN | -16.237 | -14.962 | 0.007 |
| TCGA-STAD | ENSG00000229175.1 | STAB2 | -16.237 | -14.962 | 0.007 |
| TCGA-STAD | ENSG00000229175.1 | TP53 | -16.066 | -14.819 | 0.002 |
| TCGA-STAD | ENSG00000253567.1 | - | - | - | - |
| TCGA-STAD | ENSG00000175061.13 | - | - | - | - |

The table shows somatic mutation of 16 feature lncRNAs with *P*-value <0.05. All data was downloaded from TANRIC.
