# Supplementary figures and images for: A gastric cancer LncRNAs model for MSI and survival prediction based on support vector machine
Source: BMC Genomics. 2019 Nov 13;20:846. doi: 10.1186/s12864-019-6135-x (PMC6854775; doi:10.1186/s12864-019-6135-x)

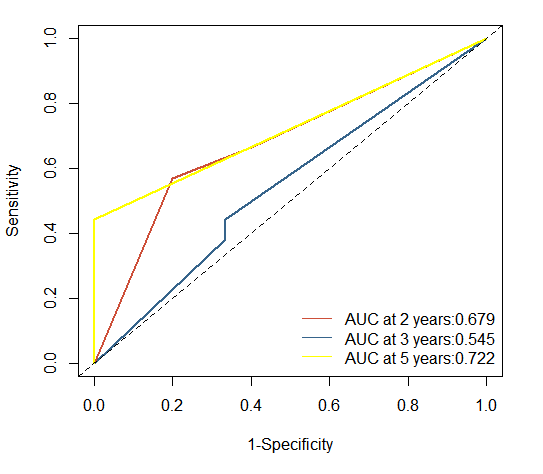

Supplement: Supplementary file 5 — Additional file 5: Figure S1. The TNM stage measured by time-dependent receiver–operating characteristic curves at 2, 3, 5 years. [file 12864_2019_6135_MOESM5_ESM.tif]

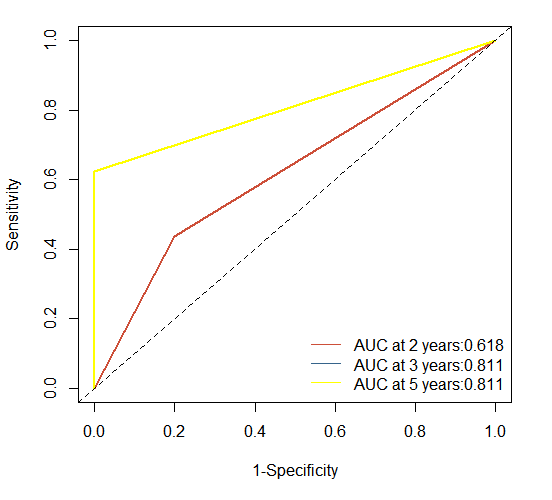

Supplement: Supplementary file 6 — Additional file 6: Figure S2. The MSI measured by time-dependent receiver–operating characteristic curves at 2, 3, 5 years. [file 12864_2019_6135_MOESM6_ESM.tif]

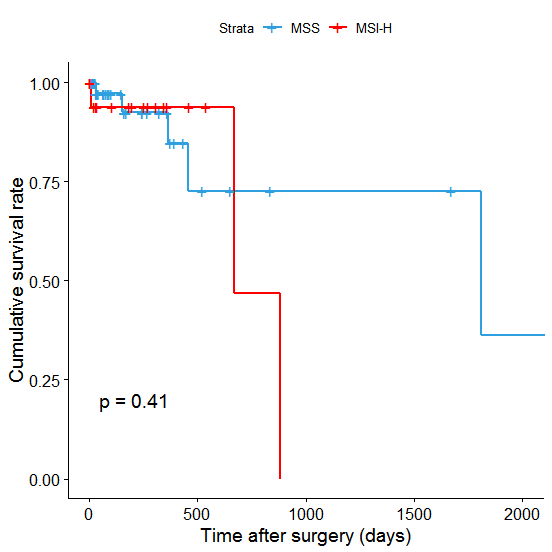

Supplement: Supplementary file 7 — Additional file 7: Figure S3. Survival impact of the MSI state. Kaplan–Meier curves for overall survival (OS) by the MSI state with patients with stage I-IV. [file 12864_2019_6135_MOESM7_ESM.tif]

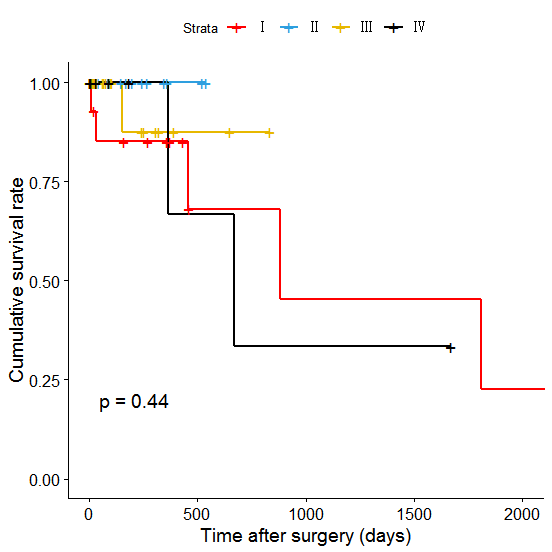

Supplement: Supplementary file 8 — Additional file 8: Figure S4. Survival impact of the TNM stage. Kaplan–Meier curves for overall survival (OS) by the TNM stage with patients with stage I-IV. [file 12864_2019_6135_MOESM8_ESM.tif]

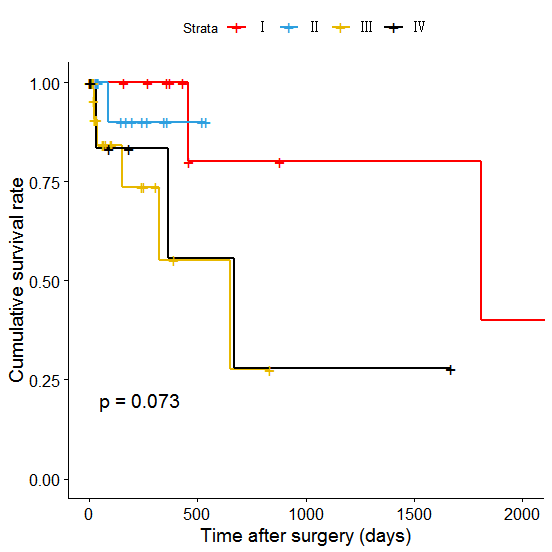

Supplement: Supplementary file 9 — Additional file 9: Figure S5. Survival impact of the TNM stage. Kaplan–Meier curves for disease-free survival (DFS) by the TNM stage with patients with stage I-IV. [file 12864_2019_6135_MOESM9_ESM.tif]
